# Raman Scattering Enhancement through Pseudo-Cavity Modes

**DOI:** 10.3390/nano14100875

**Published:** 2024-05-17

**Authors:** Vincenzo Caligiuri, Antonello Nucera, Aniket Patra, Marco Castriota, Antonio De Luca

**Affiliations:** 1 Department of Physics, University of Calabria, 87036 Rende, Italy; vincenzo.caligiuri@unical.it (V.C.); antonello.nucera@unical.it (A.N.); aniketpatra003@gmail.com (A.P.); 2 Consiglio Nazionale delle Ricerche (CNR), Istituto di Nanotecnologia (Nanotec), Sede Secondaria di Rende, 87036 Rende, Italy

**Keywords:** Raman scattering enhancement, pseudo-cavity modes, Fabry–Pérot cavities

## Abstract

Raman spectroscopy plays a pivotal role in spectroscopic investigations. The small Raman scattering cross-section of numerous analytes, however, requires enhancement of the signal through specific structuring of the electromagnetic and morphological properties of the underlying surface. This enhancement technique is known as surface-enhanced Raman spectroscopy (SERS). Despite the existence of various proposed alternatives, the approach involving Fabry–Pérot cavities, which constitutes a straightforward method to enhance the electromagnetic field around the analyte, has not been extensively utilized. This is because, for the analyte to experience the maximum electric field, it needs to be embedded within the cavity. Consequently, the top mirror of the cavity will eventually shield it from the external laser source. Recently, an open-cavity configuration has been demonstrated to exhibit properties similar to the classic Fabry–Pérot configuration, with the added advantage of maintaining direct accessibility for the laser source. This paper showcases how such a simple yet innovative configuration can be effectively utilized to achieve remarkable Raman enhancement. The simple structure, coupled with its inexpensive nature and versatility in material selection and scalability, makes it an ideal choice for various analytes and integration into diverse Raman apparatus setups.

## 1. Introduction

Raman spectroscopy is a powerful, non-destructive spectroscopic technique useful in many applications, such as the detection of pesticides, toxins, and contaminants in fruits and vegetables [1,2] the early detection of cancer biomarkers [3], microplastics detection [4], biosensing [1,5,6,7,8,9], systems of biomedical interest [10], carbon-based materials (i.e., graphene) [11,12], thin films [13], polymers [14], cultural heritage investigations [15,16,17,18,19], and many more. The precision level reached by Raman spectroscopy even extends to micro-samples through innovative techniques that involve the so-called “spectral tweezers”, a fascinating scenario that is a candidate to become a routine tool for bio-analytical investigations [20]. On the other hand, it is well known that the power of Raman investigation also extends to single-molecule spectroscopy, allowing for the non-destructive and in situ spectroscopic investigation of single particles [21]. Despite its effectiveness in all of these different kinds of investigations, Raman spectroscopy is not always performable due to the low response and/or very low concentration of the analyte. For this reason, several techniques have been recently developed to overcome such limitations, the ensemble of which goes under the name of surface-enhanced Raman spectroscopy (SERS) [22,23]. SERS allows for the detection of species with small Raman cross-sections (such as biological systems) and analytes presented in traces up to single-molecule detection [24,25,26,27,28,29,30,31]. Essentially, the weak intensity of a Raman signal coming from molecules adjacent to metallic nanostructures can be enhanced by employing the localized surface plasmon resonance (LSPR) generated in the near-field of these nanostructures [32,33,34]. Furthermore, the development of artificial material configurations (metamaterials and metasurfaces) offers advanced and unprecedented control of the interaction between the incoming electromagnetic (EM) field and biological matter, enabling the use of these plasmonic metasurfaces for sensitive SERS analysis [35,36,37]. Techniques relying on the plasmonic properties of a specifically structured metallic substrate (i.e., gold or silver), where the analyte could potentially be deposited, have largely proven their validity. The effect of photon–plasmon interaction is the promotion of Raman scattering and, consequently, of the Raman band intensities that, therefore, become more detectable. Remarkable examples of plasmonic structures for SERS are the so-called C-shaped structures [38,39,40,41]. Other examples relying on nano-fabrication techniques have been proposed with exceptional results [35,42,43,44,45,46,47]. These resonant plasmonic structures are usually fabricated through classic nano-lithography techniques that involve electron beam lithography and lift-off procedures. Plasmonic colloidal entities like bimetallic nano-stars have also proven their validity for the SERS effect [48], but functionalizing the analyte with plasmonic nanoparticles or fixing the nanoparticles over a glass substrate is often a very complicated task to accomplish. All of these drawbacks and limitations could, in principle, be simply overcome by adopting simple flat resonant structures. Using common resonant structures, like optical cavities, has long been neglected, however. For the analyte to benefit from maximum electric field enhancement, this configuration implies embedding it within the cavity, effectively shielding it from the excitation laser source through the top reflecting mirror. Examples of the use of Fabry–Pérot configuration in tip-enhanced Raman scattering (TERS) have been shown in the past, but the analyte was placed on top of the Fabry–Pérot configuration rather than inside it [49]. Recently, a new configuration has been investigated, unifying the benefits of a Fabry–Pérot resonator with an “open-cavity” geometry that allows for the embedding of the analyte within the cavity, leaving it accessible for laser source investigation [50]. Such a configuration, consisting of a simple dielectric layer on top of a metallic one, has been reported to support the so-called “pseudo-cavity” modes (PCMs) that follow a very similar dispersion with respect to the classic Fabry–Pérot one, apart from a contribution provided by the Goos–Hänchen shift, an effect directly connected to the presence of the metal/dielectric interface, according to which a beam impinging at a certain point over this interface is slightly spatially shifted due to the excitation of an evanescent wave [50].

**Figure 1:**
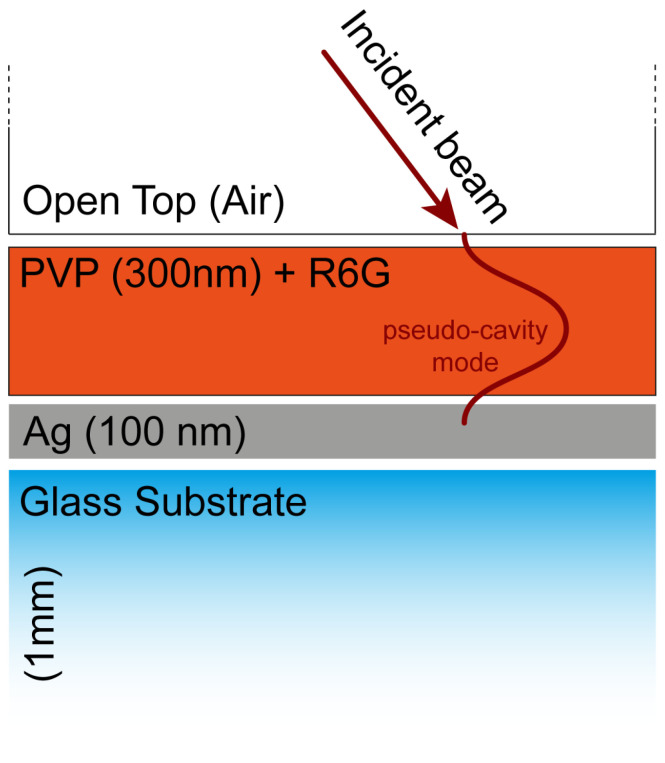
Scheme of the obtained open-cavity configuration, composed of a silver layer (100 nm) deposited on top of a glass substrate (1 mm), over which we deposited a PVP layer embedding R6 G molecules (300 nm thick). The top of the open cavity is open so that the incident laser beam impinges on the PVP+R6G layer from air.

In this work, we demonstrate that such a simple but innovative configuration results ideal for SERS applications as well. To achieve this result, we initially designed and fabricated a polyvinylpyrrolidone (PVP)-based open-cavity system with resonances tuned to the wavelength of the excitation laser source used (633 nm). We then embedded R6G molecules as a general test analyte directly into the PVP layer, so that the analyte lay directly within the open-cavity core. A sketch of the proposed system is shown in Figure 1. It can be appreciated how the Raman excitation source impinges directly on top of the PVP+R6G layer, which remains accessible to the laser source with no metallic shields or cover slip on top of it. A pseudo-cavity mode, whose electric field profile is depicted in red in Figure 1, is excited. The PCM remains mainly confined within the PVP+R6G layer so that the analyte can benefit from the large field enhancement provided by the resonance unit to the negligible reduction in the pump intensity. We demonstrate a remarkable 15-fold enhancement of the Raman signal for such a configuration, effectively switching from a no-signal to a large-signal condition, with respect to the reference sample constituted by the simple PVP+R6G layer on a glass substrate. A comparison with three additional off-resonance systems confirms that the enhancement is effectively due to the excitation of a PCM rather than simple proximity with a metallic layer. Our findings lead the way in utilizing open cavities as SERS substrates, configuring them as an inexpensive and effective universal tool for surface-enhanced Raman spectroscopy.

## 2. Materials and Methods

### 2.1. Scattering Matrix Method (SMM) Simulations

SMM simulations were carried out through a customized MatLAB R2018a code elaborating on the approach described by Rumpf et al. [51]. These calculations are based on the experimentally measured refractive index of all of the used materials as well as that of the glass substrate.

### 2.2. COMSOL-Based Simulations

COMSOL-based full-field simulations were performed via excitation with a numerical port expressing the incident electric field as a plane wave and considering the angle of incidence within the expression for the wavevector. Perfectly matched layers were properly meshed and applied. For these simulations, experimentally measured refractive indices of all of the used materials were considered as well.

### 2.3. Reflectance Measurements and Ellipsometry

P- and S-Polarized reflectance measurements, as well as spectroscopic ellipsometry experiments, were performed using the M2000 ellipsometry apparatus from Woollam, Lincoln, NE, USA.

### 2.4. Sputtering Deposition

The Ag layer was deposited via DC magnetron sputtering in Ar atmosphere (Ar pressure 4.6 × 10^−2^ mBar, power 20 W).

### 2.5. Rhodamine 6G/PVP Solution and Film Preparation

The solution was prepared by starting with a 5 wt% PVP–ethanol solution and adding a 14 mM concentration of Rhodamine 6G. Thin films were produced by spin coating the solution over a glass substrate at 3000 rpm for 30 s. In the end, a final annealing procedure (15 min at 60 °C) ensured complete solvent evaporation.

### 2.6. Raman Measurements

Raman measurements were carried out using a LABRAM Micro-Raman apparatus (Horiba Jobin-Yvon, Piscataway, NJ, USA). A 50× objective with NA = 0.7 was used. All of the measurements were carried out at full power of 17 mW with an excitation wavelength *λ* = 633 nm.

## 3. Results and Discussion

**Figure 2:**
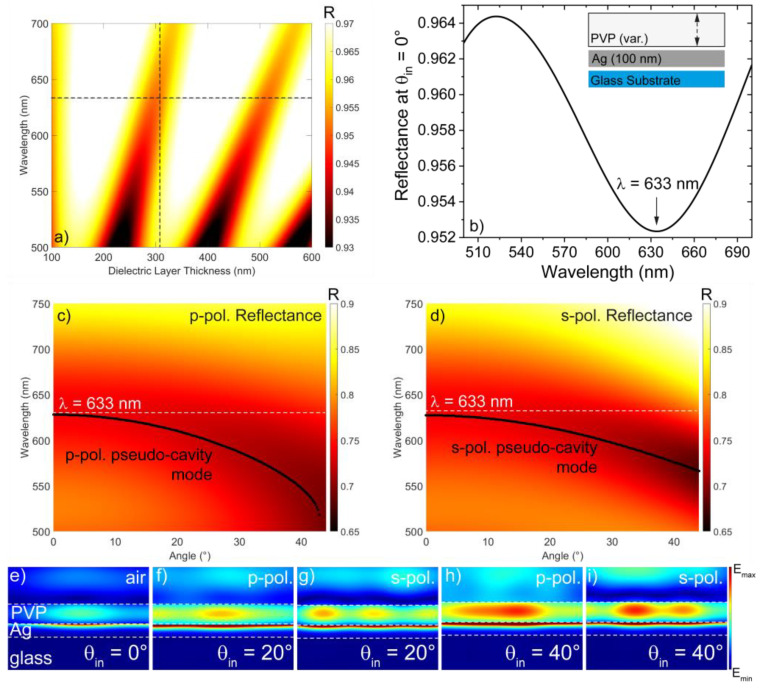
(**a**) Reflectance map at *θ*_in_ = 0°. The dashed lines are taken in correspondence with a PVP thickness of 308 nm (vertical line) and *λ* = 633 nm (horizontal). (**b**) Reflectance spectrum of the undoped Ag/PVP open-cavity system showing a minimum at *λ* = 633 nm. In the inset, a sketch of the designed open-cavity configuration. (**c**) p- and (**d**) s-polarization angular reflectance maps calculated within the acceptance cone of the objective by fixing *t_D_* = 308 nm. The precise wavelength at which the PCM is positioned is highlighted with black dots, demonstrating the blue shift as a function of the angle. The norm of the electric field was calculated through COMSOL-based simulations at (**e**) *θ* = 0°, (**f**) *θ* = 20°, p-pol., (**g**) *θ* = 20°, s-pol., (**h**) *θ* = 40°, p-pol. and (**i**) *θ* = 40°, s-pol.

The bare open-cavity system was numerically studied through the use of scattering matrix method (SMM) simulations, considering a 100 nm thick silver (Ag) bottom layer on top of a common glass substrate. The thickness of the top PVP layer was swept from 100 nm to 600 nm to obtain the dispersion map in Figure 2a. The sketch of the designed system is reported in the inset of Figure 2b. Since the Raman measurements were carried out at normal incidence through a 50× objective with a numerical aperture equal to 0.7, we can consider wavevectors spanning an angular range from 0° to about 44° impinging on the sample. Therefore, the design process was optimized for normal incidence *θ*_in_ = 0°. Pseudo-cavity modes were visible as dark bands approximately following the well-known dispersion *λ*_0_ *=* 2*n_D_t_D_/m*, where *λ*_0_ is the wavelength related to the “pseudo-cavity” mode, *n_D_* is the refractive index of the PVP layer, *t_D_* is its thickness, and *m* is the modal order [50]. A PCM was numerically found at 633 nm (wavelength of the excitation laser source) for *t_D_* ≈ 308 nm (see the crossing point between the dashed horizontal and vertical lines in Figure 2a), as confirmed by the presence of a dip at 633 nm in the reflectance spectrum extrapolated from the map in Figure 2a in correspondence with this precise PVP thickness (Figure 2b). It is worth noting that, due to both the low refractive index of PVP and the large thickness of the bottom Ag layer, the efficiency of the PCM is quite low, as evidenced by the rather high reflectance (≈0.952) manifested even in correspondence with the PCM mode. Moreover, even the Q-factor of this mode is quite low (around 6.5) as expected from this kind of mode. Remarkably, such a low Q-factor does not prevent the occurrence of the SERS effect. The resonant configuration adopted in our system introduces a well-known blue shift in the cavity mode, however, as reported in Equation (1) [50].

(1) $$\lambda \left(\theta_{i}\right)=\lambda_{0}\sqrt{1-\frac{\sin^{2}\left(\theta_{i}\right)}{n_{D}^{2}}}$$

The expression for *λ*_0_ has been provided before; *n_D_* is the refractive index of PVP and *θ_i_* is the angle of incidence that, in our case, spans the acceptance cone angular range of the objective (i.e., from 0° to 44°). To confirm that the resonance of the open-cavity system is tuned to the Raman excitation wavelength (633 nm), we investigated through SMM simulations the angular dispersion of the proposed system for both p- and s-polarization reflectance (Figure 2c,d). Here, a dashed white line highlights the Raman excitation wavelength. For both the polarizations, a blue shift in the cavity mode is visible as a dip moving toward lower wavelengths. To highlight this behavior, at each angle, the PCM was labeled with a black dot so that both the p- and s-polarization angular dispersion of the system are clearly visible, demonstrating the blue shift of the PCM. As expected, such a blue shift is quite smooth, however, with it being negligible within the first 25°. Only from 40° onward does the blue shift start to be significant, leading the resonance mode to be slightly mismatched with respect to the Raman excitation wavelength. However, this large angular range lies almost at the boundary of the acceptance cone of our experimental configuration. As a result, the excitation wavelength is matched with the cavity mode almost throughout the acceptance cone of the used objective. To confirm the validity of the open-cavity system as an SERS platform, we calculated, through COMSOL-based simulations, the modulus of the electric field at *λ* = 633 nm for normal incidence (Figure 2e) and for two additional angles within the acceptance cone of the used objective, 20° and 40°, for both p- and s-polarization (Figure 2f–i). As expected, the electric field was mostly confined within the PVP layer and at the boundary with the bottom Ag layer. Such a result confirms that by being in resonance with the open cavity, the energy of the excitation laser is mostly confined within the PVP layer as a pseudo-cavity mode. Therefore, a possibly embedded molecule could benefit from large SERS enhancement through this resonant interaction with the Raman source.

**Figure 3:**
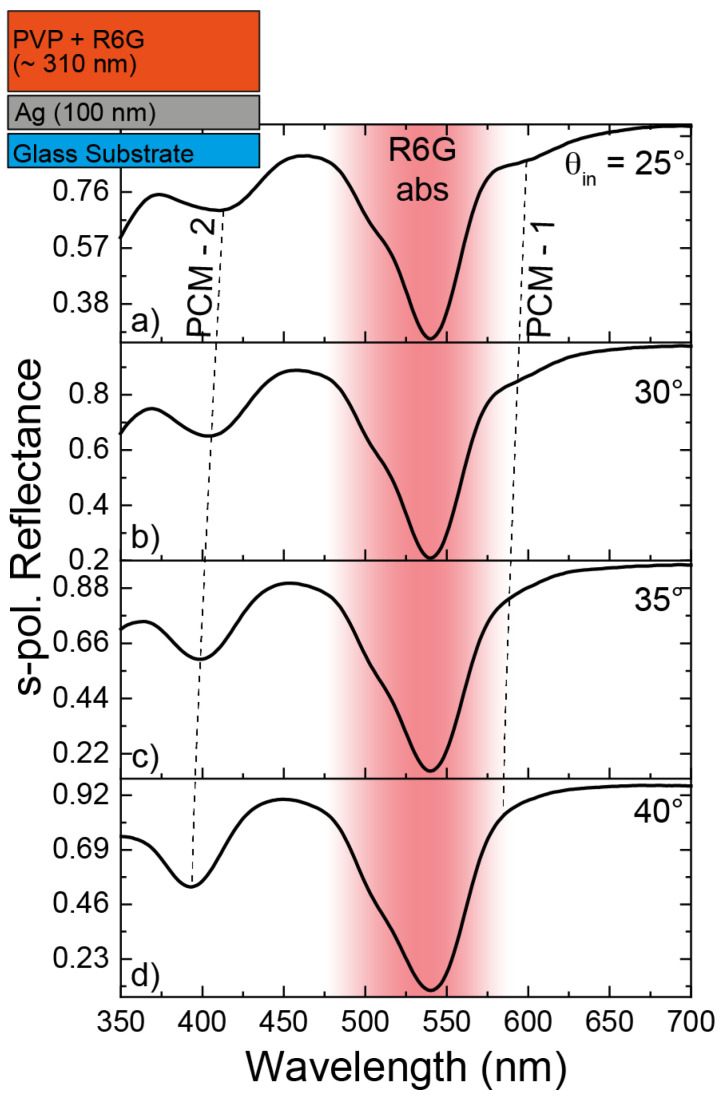
S-polarization reflectance spectra measured at *θ*_in_ equal to (**a**) 25°, (**b**) 30°, (**c**) 35°, and (**d**) 40°. In the inset, a sketch of the “pseudo-cavity” structure embedding Rhodamine 6G molecules in the PVP layer.

Rhodamine 6G molecules, selected as a benchmark analyte, were then embedded directly into the PVP layer. Since the PCM is mostly confined to the dielectric film, such a configuration maximizes the possibility of Raman enhancement. The R6G-PVP solution was obtained by adding 0.1 wt% R6G to 5 wt% PVP solution in ethanol. The solution was then spin-coated over a 100 nm thick Ag layer to build the complete dye-embedded open-cavity system sketched in the inset of Figure 3. The documented stability of the PVP at room temperature ensures the reliability of the system in laboratory conditions [52,53,54]. The s-polarization reflectance spectra, ellipsometrically measured at *θ*_in_ = 25°, 30°, 35°, and 40° (significative angles lying within the impinging wavevectors’ angular range of the Raman setup) are reported in Figure 3. The absorbance band of R6G is highlighted with a red gradient area in Figure 3. A steep dip in reflectance around ≈530 nm is evident, together with a small shoulder occurring at shorter wavelengths. These two dips, which do not shift while increasing the angle of incidence, were found to be in correspondence with the excitonic transitions of R6G [50]. Moreover, two PCMs were present: the first one, labeled as PCM-1, occurs at about 600 nm for a *θ*_in_ = 25° and represents the one of interest for our investigation. The second one, labeled as PCM-2, occurs at a shorter wavelength and corresponds to the successive harmonics. Even though PCM-2 does not play any role in Raman enhancement, its spectral features are more recognizable than those of PCM-1, with this mode being far from the absorbance band of R6G. PCM-2 manifests a marked blue shift while increasing the angle of incidence, respecting the PCM’s angular dispersion reported in Equation (1).

PCM-1 follows the same angular dynamics, as highlighted by the dashed lines in Figure 3, which show the spectral shifts of the two modes. Therefore, it is possible to state that, at angles smaller than 25° (angles inaccessible with our spectroscopic setup), PCM-1 red-shifts, finally resulting in being in tune with the wavelength of the excitation source (633 nm) at normal incidence.

**Figure 4:**
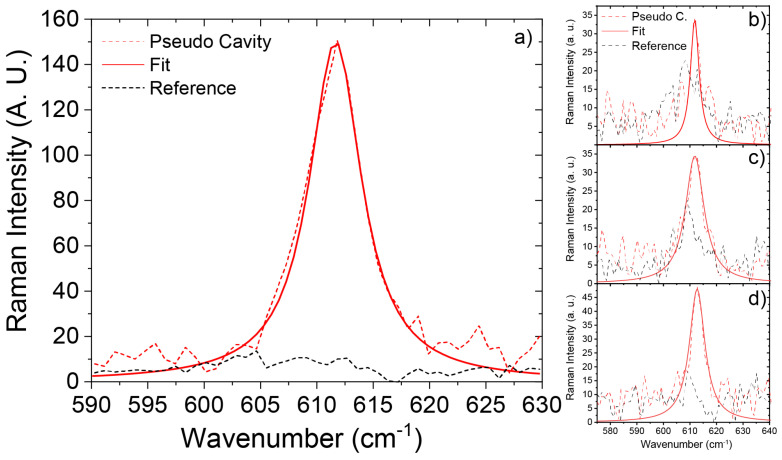
Comparison between the Raman signal in the pseudo-cavity configuration (dashed red curve) and the reference sample (bare PVP+R6G—dashed black curve) in the spectral range of the xanthene ring of R6G molecules, measured for different samples obtained at four different spin-coating speeds: (**a**) 2000 rpm (pseudo-cavity mode tuned to the Raman laser source wavelength), (**b**) 2500 rpm, (**c**) 3000 rpm, and (**d**) 3500 rpm.

Raman measurements were then carried out on four different systems, where the thickness of the PVP+R6G layer was tuned in- and off-resonance by increasing the spin coating speed. To evaluate the enhancement of the Raman signal, we focused our attention on the peak at about 611 cm^−1^, associated with the deformation of the xanthene ring of R6G molecules. The first system was obtained by spin-coating a PVP+R6G film at 2000 rpm speed over the Ag layer, resulting in a pseudo-cavity mode tuned to the Raman excitation wavelength with the thickness of the PVP+R6G layer being about 310 nm (ellipsometrically measured). Three other cases were evaluated: (i) 2500 rpm, (ii) 3000 rpm, and (iii) 3500 rpm, with all of them leading to thinner dielectric layers with blue-shifted PCMs, resulting in off-resonance systems. Each of the four systems was compared with a related reference sample consisting of the bare PVP+R6G layer, spin-coated at equivalent speed directly on the glass substrate. For the first case, a 27-fold enhancement of the Raman signal around ≈611 cm^−1^ was found (Figure 4a), confirming the beneficial role played by the pseudo-cavity mode. Measurements carried out over the three additional off-resonance systems (Figure 4b–d) revealed Raman intensity comparable to that of the related reference systems. It is worth noting that a paltry feature related to the Raman shift of the xanthene at ≈611 cm^−1^ is visible even in the references of Figure 4b,c. This is due to R6G agglomeration in the specific point of investigation. Such a result not only confirms that the enhancement found for the first system is ascribable to the pseudo-cavity mode but also rules out the possibility that it is solely due to the simple effect of the underlying Ag layer. We can, however, compare the signal acquired for the resonant system (red curve Figure 4a) with those related to the R6G aggregates in the references of Figure 4b,c to put ourselves in the worst condition. We still observed a remarkable 7.5-fold enhancement.

## 4. Conclusions

In conclusion, in this work, we demonstrate that PCMs occurring in an open-cavity system can offer an easy and effective pathway to provide exceptional enhancement of the Raman signal. The main result was achieved by engineering an open-cavity configuration whose associated PCM was tuned to the excitation laser wavelength of a Raman system. A 27-fold enhancement of Raman intensity was found, enabling the detection of a previously undetectable feature. We proved the effect of the PCM over three additional systems, whose resonances were specifically detuned with respect to the excitation wavelength, resulting in a practically negligible effect on the Raman signal. Our findings pioneer the use of open-cavity architectures as Raman-enhancing substrates. The proposed systems, assuming the shape of a simple glass microscope slab, can be readily proposed as stand-alone substrates to be integrated into every Raman microscopy system, offering a cheap solution for enhanced Raman scattering measurements. Perspectives for future improvements involve the investigation of similar effects via simple deposition on top of solid-state open-cavity systems and the investigation of numerous additional analytes.

## Data Availability

The produced data are available from the authors upon reasonable request.
